# Role of Cathepsin S in Periodontal Inflammation and Infection

**DOI:** 10.1155/2017/4786170

**Published:** 2017-12-06

**Authors:** S. Memmert, A. Damanaki, A. V. B. Nogueira, S. Eick, M. Nokhbehsaim, A. K. Papadopoulou, A. Till, B. Rath, S. Jepsen, W. Götz, C. Piperi, E. K. Basdra, J. A. Cirelli, A. Jäger, J. Deschner

**Affiliations:** ^1^Section of Experimental Dento-Maxillo-Facial Medicine, Center of Dento-Maxillo-Facial Medicine, University of Bonn, Bonn, Germany; ^2^Department of Orthodontics, Center of Dento-Maxillo-Facial Medicine, University of Bonn, Bonn, Germany; ^3^Department of Diagnosis and Surgery, School of Dentistry at Araraquara, Sao Paulo State University (UNESP), Araraquara, SP, Brazil; ^4^Department of Periodontology, Laboratory for Oral Microbiology, School of Dental Medicine, University of Bern, Bern, Switzerland; ^5^Discipline of Orthodontics, Faculty of Dentistry, University of Sydney, Sydney, NSW, Australia; ^6^Institute of Reconstructive Neurobiology, Life & Brain Center, University of Bonn, Bonn, Germany; ^7^Department of Periodontology, Operative and Preventive Dentistry, University of Bonn, Bonn, Germany; ^8^Department of Biological Chemistry, Medical School, National and Kapodistrian University of Athens, Athens, Greece; ^9^Noel Martin Visiting Chair, Faculty of Dentistry, University of Sydney, Sydney, NSW, Australia

## Abstract

Cathepsin S is a cysteine protease and regulator of autophagy with possible involvement in periodontitis. The objective of this study was to investigate whether cathepsin S is involved in the pathogenesis of periodontal diseases. Human periodontal fibroblasts were cultured under inflammatory and infectious conditions elicited by interleukin-1*β* and *Fusobacterium nucleatum*, respectively. An array-based approach was used to analyze differential expression of autophagy-associated genes. Cathepsin S was upregulated most strongly and thus further studied *in vitro* at gene and protein levels. *In vivo*, gingival tissue biopsies from rats with ligature-induced periodontitis and from periodontitis patients were also analyzed at transcriptional and protein levels. Multiple gene expression changes due to interleukin-1*β* and *F. nucleatum* were observed *in vitro*. Both stimulants caused a significant cathepsin S upregulation. A significantly elevated cathepsin S expression in gingival biopsies from rats with experimental periodontitis was found *in vivo*, as compared to that from control. Gingival biopsies from periodontitis patients showed a significantly higher cathepsin S expression than those from healthy gingiva. Our findings provide original evidence that cathepsin S is increased in periodontal cells and tissues under inflammatory and infectious conditions, suggesting a critical role of this autophagy-associated molecule in the pathogenesis of periodontitis.

## 1. Introduction

Periodontitis is currently the most common chronic inflammatory disease with a prevalence of over 50% in patients over 30 years of age [1]. It is characterized by the irreversible destruction of the periodontium and is the primary reason for tooth loss in adult patients. The periodontium comprises the tooth-supporting tissues, that is, the gingiva, periodontal ligament (PDL), root cementum, and alveolar bone. PDL is of heterogeneous nature with fibroblasts constituting the dominant cell type and displaying a major role in extracellular matrix synthesis and remodeling [2]. However, not only are PDL cells crucial in the maintenance of periodontal homeostasis, but they also play a pivotal role in the local immune response [3].

Periodontal destruction is initiated by microorganisms present in the subgingival plaque, such as *Fusobacterium nucleatum* [4, 5]. During the development of periodontitis, the pathogenic bacteria lead to an inflammatory host response, resulting in the synthesis of inflammatory mediators, such as interleukin- (IL-) 1*β*. The interplay of destructive microorganisms and host response can elicit matrix degradation and bone resorption. However, the aetiology of periodontitis is multifactorial, and different cofactors, such as smoking, genetic factors, and occlusal loading, are also critical for the initiation and progression of the disease. Pocket formation and resultant tooth loss are not the only consequences of untreated periodontitis [6, 7], as definite links exist between periodontitis and a number of other diseases related to general health, such as diabetes mellitus, myocardial infarction, and stroke [8, 9].

Autophagy is a highly conserved mechanism of cellular homeostasis through self-consumption and is important for the response to intrinsic and extrinsic insults, such as malnutrition and mechanical, oxidative, or inflammatory stressors [10–12]. In such an event and according to the threshold reached by the stressors, autophagy can either secure cell survival or lead to cell death [13]. Furthermore, autophagy plays an important role in removing misfolded proteins, nonfunctional organelles, and intracellular pathogens [14]. Three types of autophagy are described in eukaryotic cells: macroautophagy (referred to as “autophagy” in this paper), microautophagy, and chaperone-mediated autophagy [14]. Autophagy-dependent mechanisms are fundamental in the maintenance of the inflammatory balance as documented by the involvement in the pathogenesis of inflammatory disorders, such as diabetes mellitus and inflammatory bowel disease [15, 16]. Interestingly, increased mRNA and protein levels of autophagy-related genes (ATGs) have been found in peripheral blood mononuclear cells from patients with periodontitis [17], suggesting that autophagy may also play a role in the pathogenesis of periodontitis. However, the exact mechanism of how autophagy is part of PDL cell immune responses to inflammatory and microbial challenges is still unknown.

ATGs have been identified as key regulators of autophagic responses in cells. Over 30 different ATGs have been identified to participate in the molecular control of autophagy [18, 19]. However, additional molecules are involved in this intracellular degradation process. For example, the inhibition of cathepsin S (CTSS) has been found to induce autophagy through reactive oxygen species- (ROS-) mediated phosphatidylinositol 3-kinase (PI3K) and c-Jun N-terminal kinase (JNK) signaling pathways [20, 21]. CTSS is a lysosomal multifunctional cysteine protease, and its intracellular functions include the processing of proteins during trafficking and secretion. Interestingly, CTSS is not expressed ubiquitously but shows a restricted pattern of distribution [22]. It is produced by immune cells like macrophages, microglia, B-lymphocytes, and dendritic cells and is shown to be induced by inflammatory insults [23, 24]. CTSS is also important during antigen processing and presentation, as it breaks down antigenic as well as antimicrobial peptides [25–27]. Extracellularly, CTSS remains stable and active under neutral pH values and functions as an elastase able to destroy extracellular matrix proteins, such as collagen, as well as proteins of the bacterial outer membrane [25, 28]. CTSS is closely related to cathepsin K (CTSK), which has been shown to be upregulated in periodontitis [25, 29]. Evidence from a bioinformatics screen that compared the gene expression profile between patients with periodontitis and normal controls has identified the likely involvement of CTSS in periodontitis [30].

Despite the presence of some links between CTSS and oral inflammatory diseases, the definite role of CTSS has yet to be unraveled. Elucidation of the involvement of CTSS in the regulation of inflammatory and microbial threats in PDL cells might be an approach to further clarify the role of autophagy in periodontitis. Our hypothesis was that microbial and inflammatory stimuli would impact CTSS levels in periodontal cells and tissues, thereby suggesting a critical role of this autophagy-associated molecule in the pathogenesis of periodontal diseases.

## 2. Materials and Methods

### 2.1. Isolation and Characterization of PDL Cells

After the approval of the Ethics Committee of the University of Bonn and written informed consent by the subjects (#117/15), PDL cells were harvested from periodontal healthy donors (mean age: 14.1 years, min–max: 11–19 years), who underwent tooth extractions for orthodontic reasons as described elsewhere [31, 32]. In order to avoid contamination from gingival, pulpal, and bone cells, PDL cells were explanted from the medial part of the tooth root and cultured in Dulbecco's minimal essential medium (DMEM, Invitrogen, Karlsruhe, Germany) supplemented with 10% fetal bovine serum (FBS, Invitrogen), 100 units/mL penicillin, and 100 *μ*g/mL streptomycin (Invitrogen) at 37°C in a humidified atmosphere of 5% CO_2_. After phenotyping with known specific markers [32], PDL cells (3rd to 5th passage) were seeded (50,000 cells/well) on 6-well plates and grown to 80% confluence.

### 2.2. Cell Stimulation

One day prior to stimulation, the FBS concentration was reduced to 1% to minimize interactions with its components. In order to simulate inflammatory or infectious conditions *in vitro*, cells were exposed to IL-1*β* (PromoKine, Heidelberg, Germany; 0.1–10 ng/mL) or to the periodontopathogen *F. nucleatum* (ATCC 25586; optical density: 0.0125–0.050). The inactivation of the bacteria was achieved by suspension in PBS (OD_660 nm_ = 1, equivalent to bacterial cells/mL) and ultrasonication (160 W twice for 15 min). To provide comparable results, IL-1*β* and *F. nucleatum* were utilized at the same physiological concentrations as in our previous studies [33–38]. Cells cultured without any stimuli served as controls. Intracellular signaling employed in the activation of CTSS by inflammatory or infectious stimuli was examined by the usage of specific inhibitors (all from Calbiochem, San Diego, CA, USA; 10 *μ*M) against the MEK1/2 (U0126), JNK (SP600125), p38 (SB203580), and NF-*κ*B (PDTC) signaling pathways. Preincubation of cells with inhibitors was performed 1 h prior to experiments.

### 2.3. Analysis of Gene Expression

After the cells of 3 donors were exposed to inflammatory and infectious stimuli for 24 h or left untreated, total RNA was extracted with a commercially available RNA extraction kit (RNeasy Protect Minikit, Qiagen, Hilden, Germany). A total of 1 *μ*g of RNA was reverse-transcribed for cDNA synthesis with the iScript™ Select cDNA Synthesis Kit (Bio-Rad Laboratories, Munich, Germany) and used in the PrimePCR™ Assay. To analyze variations in the expression of autophagy-associated genes, a specific PrimePCR Assay (Autophagy (SAB Target List) H96, Bio-Rad Laboratories) was applied in accordance with the manufacturer's instructions. A list of the genes covered by this assay is available at the manufacturer's website (http://www.bio-rad.com/de-de/prime-pcr-assays/predesigned-plate/sybr-green-autophagy-sab-target-list-h96). For real-time polymerase chain reaction (RT-PCR), an iCycler iQ5 Detection System (Bio-Rad Laboratories) was used. For data normalization, three reference genes (glyceraldehyde-3-phosphate dehydrogenase (GAPDH), hypoxanthin-phosphoribosyl-transferase 1 (HPRT1), and TATA box-binding protein (TBP)) were embedded in the plate setup and employed for comparative ΔΔ-CT analysis with the Software CFX-Manager (Bio-Rad Laboratories) as provided by the manufacturer.

For validation of regulated CTSS expressions, RT-PCR was conducted with 1 *μ*L of cDNA in 25 *μ*L reaction mixture containing 2.5 *μ*L respective QuantiTect Primer Assay (Qiagen), 12.5 *μ*L QuantiTect SYBR Green Master Mix (Qiagen), and 9 *μ*L nuclease-free water. The applied protocol consisted of a heating phase at 95°C for 5 min to activate the enzyme, 40 cycles including a denaturation step at 95°C for 10 s, and a combined annealing/extension step at 60°C for 30 s per cycle. Melting point analysis was performed after each run. GAPDH was determined for normalization as a housekeeping gene. Further analysis of CTSS gene expression was carried out accordingly.

### 2.4. ELISA

The commercially available enzyme-linked immunosorbent assay (ELISA) kit (RayBiotech, Norcross, GA, USA) was used to measure released protein amounts of CTSS in cell-free supernatants after inflammatory or infectious PDL cell stimulation for 24 h and 48 h. The manufacturer's instructions were followed precisely, and absorbance was measured with a microplate reader (PowerWave X, BioTek Instruments, Winooski, VT, USA) at 450 nm. Data normalization was achieved according to the cell number as determined with an automatic cell counter (Moelab, Hilden, Germany).

### 2.5. Immunocytochemistry

PDL cell stimulation with IL-1*β* (1 ng/mL) and *F. nucleatum* (OD: 0.025) was performed as described above, and cells were cultured on glass coverslips (Carl Roth, Karlsruhe, Germany) in 24-well plates for 24 h. Cells were prepared for immunocytochemistry by fixation in 4% paraformaldehyde (Sigma-Aldrich, Munich, Germany) at pH 7.4 and room temperature and by permeabilization with 0.1% Triton X-100 (Sigma-Aldrich). Each incubation step was followed by two washing steps with PBS (Sigma-Aldrich). To block unspecific background staining, cells were suspended in serum block (Dako, Hamburg, Germany) for 20 min, followed by incubation with rabbit polyclonal antibody anti-CTSS (Abcam, Cambridge, MA, USA; 1 : 250) at 4°C overnight. Cells were then labeled with a goat anti-rabbit IgG-HRP secondary antibody (Dako) for 45 min. Antibody binding was made visible by DAB chromogen (Thermo Fisher Scientific, Waltham, MA, USA) staining for 10 min at room temperature. Counterstaining with Mayer's hematoxylin (Merck, Darmstadt, Germany) for 1 min was followed, and finally, coverslips were mounted with DePex mounting medium (SERVA Electrophoresis, Heidelberg, Germany). An Axioskop 2 microscope (Carl Zeiss, Jena, Germany) with an AxioCam MRc camera (Carl Zeiss) and the AxioVision 4.7 software (Carl Zeiss) were used for standardized imaging.

### 2.6. Experimental Periodontitis Model

To study the expression of CTSS in gingival biopsies during the development of periodontal disease, a rat model was used [39]. In total, 24 rats were used and assigned randomly to two experimental groups: control (sham-operated) and ligature-induced periodontal disease. Periodontitis was induced in male adult Holtzman rats (average weight: 300 g) through cotton ligatures placed around the cervical area of the upper first molars. They were knotted mesially and left in place for 6, 8, and 12 d. The approval of the Ethical Committee on Animal Experimentation (protocol number: 23/2012) from the School of Dentistry at Araraquara, São Paulo State University (UNESP), was given, and the protocol followed the ARRIVE guidelines. Animals were kept in plastic cages in the animal facilities of the School of Dentistry at Araraquara under controlled thermal conditions of 22–25°C with a 12 h light/dark cycle. They were fed with a standard laboratory diet and received water ad libitum. General anesthesia was given with intramuscular injections of 10% ketamine hydrochloride (0.08 mL/100 g body weight) and 2% xylazine hydrochloride (0.04 mL/100 g body weight). After the assigned points of time, 4 animals per group were sacrificed by an anesthetic overdose. Their maxillary jaws were collected, and gingival tissues around the maxillary first molars were dissected for extraction of total RNA for RT-PCR (see above).

### 2.7. Human Biopsies

The human gingivae of 7 periodontally healthy donors (mean age: 22.1 years, min–max: 18–26 years; gender: 2 males/5 females) and 7 patients with diagnosed periodontitis (mean age: 58.4 years, min–max: 29–81 years; gender: 5 males/2 females) were collected in the Department of Oral Surgery of the University of Bonn during wisdom tooth extractions or extractions of teeth for orthodontic or periodontal reasons [37]. Written informed consent and approval of the Ethics Committee of the University of Bonn were obtained (#043/11). Gingival sites were classified as clinically periodontally healthy or diseased by the gingival index (GI), probing pocket depth (PD), clinical attachment level (CAL), and radiographic bone loss. Sites with GI = 0 (no clinical inflammation), PD ≤ 3 mm, and neither clinical nor radiographic bone loss were graded as healthy. Sites with GI > 1, PD ≥ 5 mm, and clinical as well as radiographic bone loss ≥ 3 mm were categorized as periodontally diseased. Smoking, the use of medication, and presence of systemic diseases were the criteria of exclusion. RNA extraction and RT-PCR were carried out as described above. In addition, gingival biopsies were fixed in 4% phosphate-buffered paraformaldehyde (Sigma-Aldrich) for 48 h. After fixation, hydration, and dehydration, tissues were embedded in paraffin (McCormick Scientific, Richmond, IL, USA). Serial sections of 2.5 *μ*m thickness were obtained, and tissues were mounted on glass slides (Carl Roth) and allowed to dry at 37°C overnight. Immunohistochemistry analysis of CTSS was performed on deparaffinized and rehydrated tissue sections after washing with TBS (components: TRIS, MP Biomedicals, Illkirch, France, and NaCl, Merck) for 10 min. To block endogenous peroxidase, 0.3% methanol (AppliChem, Darmstadt, Germany)/H_2_O_2_ (Merck) solution was used in the dark for 10 min. Then, sections were incubated with a rabbit polyclonal antibody to CTSS (Abcam; 1 : 200) in a humid chamber at 4°C overnight. Subsequently, sections were rinsed and incubated with a goat anti-rabbit IgG-HRP secondary antibody (Dako) at room temperature for 30 min. After another washing step, antibody binding was visualized using DAB chromogen (Thermo Fisher Scientific) for 10 min. Slides were rinsed, counterstained with Mayer's hematoxylin (Merck) and coverslipped for analysis. Standardized images were taken with an Axioskop 2 microscope (Carl Zeiss) with an AxioCam MRc camera and the AxioVision 4.7 software (Carl Zeiss).

### 2.8. Statistical Analysis

The IBM SPSS Statistics software (Version 22, IBM SPSS, Chicago, IL, USA) was used for statistical analysis. Mean values and standard errors of the mean (SEM) were calculated for quantitative data. All experiments were performed in triplicate and repeated at least twice. For statistical comparison of the groups, the *t*-test, ANOVA followed by the posthoc Dunnett and Tukey tests, and the Mann-Whitney *U* test were applied. Differences between groups were considered significant at *p* < 0.05.

## 3. Results

### 3.1. Effects of Inflammatory and Infectious Stimulants on Autophagy-Related Target Genes

First, we analyzed the effects of inflammatory and infectious stimulants on the gene expression of targets known to be involved in autophagy regulation by a microarray-based approach. To mimic an inflammatory or infectious environment, PDL cells of three different donors were incubated with either IL-1*β* or *F. nucleatum* for 1 d. As shown in Tables 1 and 2, both stimulants caused regulatory effects on multiple targets: IL-1*β* caused an upregulation of CTSS and DNA damage-regulated autophagy modulator 1 (DRAM1) as well as a downregulation of B-cell lymphoma 2 (BCL2); autophagy-related gene 3 (ATG3), tumor necrosis factor receptor superfamily, member 6 (FAS); transmembrane protein 74 (TMEM74); death-associated protein kinase 1 (DAPK1); eukaryotic translation initiation factor 4 gamma (ESR1); CXC motif, receptor 4 (CXCR4); and immunity-related GTPase family M (IRGM). *F. nucleatum* increased the expression of CTSS; phosphoinositide-3-kinase, catalytic, gamma polypeptide (PIK3CG); DRAM1; insulin-like growth factor 1 (IGF1); nuclear factor of kappa light polypeptide gene enhancer in B-cells 1 (NFKB1); BH3 interacting domain death agonist (BID); BCL2 antagonist killer 1 (BAK1); tumor necrosis factor (ligand) superfamily, member 10 (TNFSF10); insulin (INS); and TMEM74.

### 3.2. Stimulation of CTSS Synthesis by IL-1*β* and *F. nucleatum*

**Figure 1:**
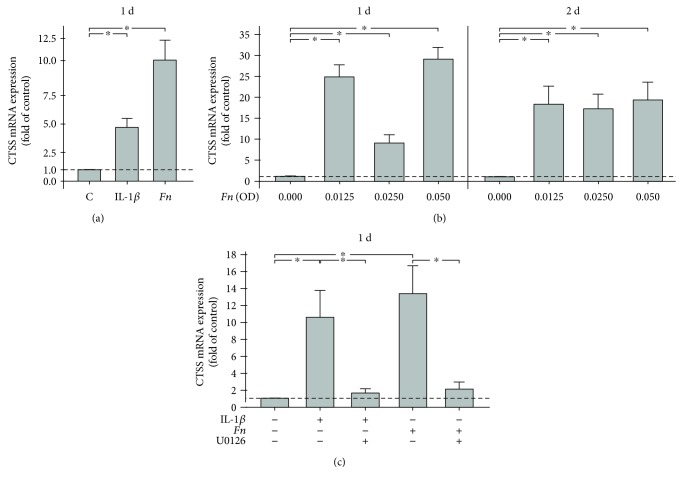
(a) Effects of IL-1*β* (1 ng/mL) or *F. nucleatum* (OD: 0.025) on CTSS mRNA expression at 1 d. Untreated cells served as the control. Mean ± SEM (*n* = 9); ^∗^significant (*p* < 0.05) difference between groups. (b) Effects of various concentrations of *F. nucleatum* (OD: 0.00–0.05) on CTSS mRNA expression at 1 d and 2 d. Mean ± SEM (*n* = 9); ^∗^significant (*p* < 0.05) difference between groups. (c) Effects of IL-1*β* (1 ng/mL) or *F. nucleatum* (OD: 0.025) on CTSS mRNA expression in cells either preincubated or not with a specific MEK1/2 inhibitor (U0126) at 1 d. Untreated cells served as the control. Mean ± SEM (*n* = 6); ^∗^significant (*p* < 0.05) difference between groups.

The protease CTSS was the target upregulated most strongly by both stimulants and thus chosen for subsequent validation by RT-PCR. Confirming the results of the array, IL-1*β* and *F. nucleatum* enhanced significantly the CTSS mRNA levels in PDL cells by 4.70-fold and 10.18-fold, respectively, as demonstrated in Figure 1(a). Furthermore, we studied the dose and time response of PDL cells to both stimulants. As depicted in Figure 1(b), the stimulatory effects of IL-1*β* and *F. nucleatum* were observed over 2 d and a wide range of concentrations. Similar to *F. nucleatum*, a significant CTSS upregulation was also found for different concentrations of IL-1*β* at both time points (data not shown).

To analyze how the effects of both stressors are conveyed, PDL cells were incubated with inhibitors against the MEK1/2 (U0126) and JNK (SP600125) signaling pathways prior to the stimulation of cells with IL-1*β* and *F. nucleatum*. Interestingly, preincubation of cells with the specific inhibitor against the MEK1/2 pathway abrogated significantly the stimulatory effects of IL-1*β* and *F. nucleatum* on the CTSS expression at 1 d (Figure 1(c)). Similarly, the CTSS upregulation by both stimulants was significantly inhibited by preincubation of cells with the specific JNK inhibitor, which reduced the IL-1*β*- and *F. nucleatum*-upregulated CTSS expressions by 45% and 73%, respectively. Inhibitors against p38 (SB203580) and NF-*κ*B (PDTC) signaling pathways had no significant effect on the CTSS expression (data not shown).

**Figure 2:**
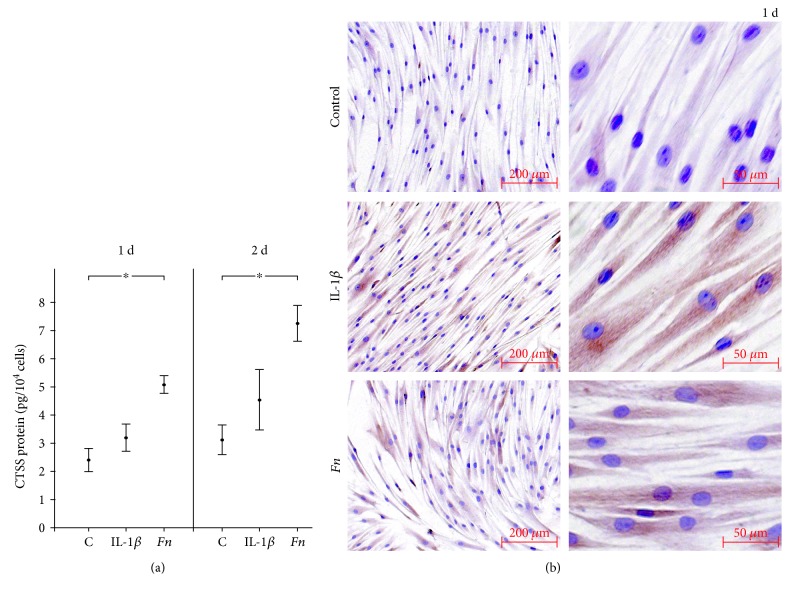
(a) Effects of IL-1*β* (1 ng/mL) or *F. nucleatum* (OD: 0.025) on CTSS protein level in supernatants at 1 d and 2 d, as determined by ELISA. Untreated cells served as the control. Mean ± SEM (*n* = 12); ^∗^significant (*p* < 0.05) difference between groups. (b) Effects of IL-1*β* (1 ng/mL) or *F. nucleatum* (OD: 0.025) on CTSS protein level in cells at 1 d, as analyzed by immunocytochemistry. Untreated cells served as the control. Experiments were performed in triplicates, and representative images of cells from one donor are shown.

The changes in CTSS gene expression by IL-1*β* and *F. nucleatum* were further studied at protein level. As analyzed by ELISA, *F. nucleatum* increased significantly the CTSS protein level in cell supernatants at 1 d and 2 d (Figure 2(a)). Similarly, IL-1*β* also led to enhanced CTSS protein levels in supernatants at both days, even though the differences did not reach significance due to the biological variations among cells. The elevated CTSS production by PDL cells was also evidenced by immunocytochemistry. IL-1*β* caused a distinct increase in CTSS protein levels in the cell cytoplasm (Figure 2(b)). Although less pronounced, PDL cells exposed to *F. nucleatum* also showed enhanced staining for CTSS (Figure 2(b)).

### 3.3. CTSS Synthesis in Rat and Human Gingival Biopsies

**Figure 3:**
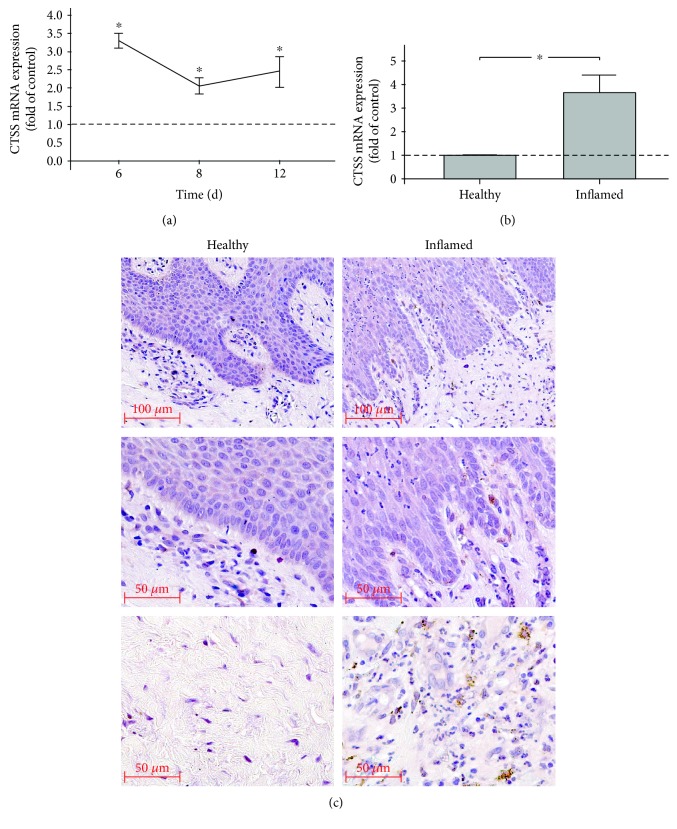
(a) CTSS mRNA expression in gingival biopsies of rats with ligature-induced experimental periodontitis at 6, 8, and 12 d, as compared to that of healthy controls. Mean ± SEM (*n* = 4 per group and time point); ^∗^significant (*p* < 0.05) difference between groups. (b) CTSS mRNA expression in human gingival biopsies from periodontally healthy and periodontitis sites. Mean ± SEM (*n* = 7 per group); ^∗^significant (*p* < 0.05) difference between groups. (c) CTSS protein in human gingival biopsies from periodontally healthy or periodontitis sites, as revealed by immunohistochemistry. Representative images of biopsies from one healthy individual and one periodontitis patient are shown.

*In vivo*, the regulation of CTSS expression in experimental periodontitis was followed in a rat model over 2 weeks. Periodontitis was established by cotton ligatures and evidenced by periodontal inflammation and alveolar bone loss, as previously shown [39]. As analyzed by RT-PCR, CTSS expressions were significantly elevated at 6, 8, and 12 d (Figure 3(a)).

In addition, human gingival biopsies from sites of periodontitis were compared with gingival tissues from periodontally healthy sites [37]. Analysis by RT-PCR revealed that CTSS expression levels were significantly higher at sites of periodontitis, as compared to control sites (Figure 3(b)). Furthermore, CTSS protein was also detected in gingival biopsies from periodontally diseased sites by immunohistochemistry. A strong immunoreaction was found in fibroblasts of the gingiva propria as well as in macrophages of the inflammatory cell infiltrate but not in epithelial cells (Figure 3(c)). No staining for CTSS was observed in biopsies from healthy sites (Figure 3(c)).

## 4. Discussion

The present study provides novel evidence that CTSS is expressed in human fibroblast-like PDL cells and upregulated under inflammatory and infectious conditions. Our observations were further supported by findings from gingival biopsies retrieved from both patients affected with periodontitis and mammals subjected to experimental periodontal disease. Therefore, CTSS may be an important player in the initiation and progression of periodontal diseases. Since CTSS is strongly associated with autophagy, our results suggest the involvement of autophagic processes in the pathogenesis of periodontitis.

CTSS shows a restricted pattern of distribution limited mostly to professional antigen-presenting cells like dendritic and B-cells [40]. Several functions of CTSS have been revealed, such as the involvement in major histocompatibility complex (MHC) class II maturation and antigen presentation [40]. Moreover, CTSS can degrade antimicrobial peptides through cleavage and thereby lead to impaired antimicrobial activity [27]. In inflamed synovia, CTSS participates in matrix degradation [41]. Further studies have confirmed the catabolic role of CTSS as a potent elastinolytic enzyme capable of degrading fibronectin, laminin, and myelin basic protein and proteoglycans [25]. Additionally, CTSS also affects osteoblast differentiation and bone remodeling [42]. Several studies have also demonstrated that CTSS functions as a critical regulator of autophagy [20, 43]. Furthermore, CTSS is implicated in osteoimmunological diseases, such as rheumatoid arthritis, as well as in vascular and metabolic complications of obesity, like diabetes mellitus [41, 42, 44, 45]. Interestingly, these diseases have all been shown to be significantly associated with periodontal diseases [8, 46, 47]. Taken together, CTSS is characterized by a plethora of actions in immunomodulatory and catabolic processes.

Inflammatory and infectious insults led to an altered expression of CTSS and other autophagy-associated molecules in our experiments, as revealed by a PCR array, that is, an unbiased approach. Since CTSS was detected as the most strongly upregulated molecule, we further focused on this multifunctional lysosomal cysteine protease. The upregulation of CTSS expression could be confirmed by RT-PCR and was also observed at protein level, as analyzed by ELISA and immunocytochemistry. To establish an inflammatory environment in the *in vitro* experiments, IL-1*β* was used as in our previous studies [33–37]. This potent inflammatory mediator has been shown to be increased at inflamed gingival sites. The anaerobic microorganism *F. nucleatum* was used to simulate periodontal microbial infection. This microorganism is one of the first gram-negative bacteria to colonize the oral biofilm and considered a “bridge organism” because of its ability to support colonization of other bacteria [48, 49]. Although both stimulants significantly upregulated the CTSS expression, as determined by RT-PCR, only *F. nucleatum* caused significantly increased protein levels in supernatants, as measured by ELISA. Nevertheless, higher CTSS levels were also found in IL-1*β*-stimulated PDL cells, as compared to unstimulated control cells, even if the differences did not reach significance. By contrast, the staining for CTSS protein, as visualized by immunocytochemistry, was more pronounced in the IL-1*β*-stimulated cells, as compared to cells incubated with *F. nucleatum*. These discrepancies could point at different roles of CTSS in the presence of inflammatory or infectious insults. For example, it is conceivable that CTSS plays a more extracellular role in microbial infections through its bactericidal activity against bacteria, as already shown for other cathepsins [50, 51]. In contrast, CTSS induced by IL-1*β* might exert more lysosomal activities, which could explain the lower CTSS levels in supernatants and the more pronounced staining of the cytoplasm of stimulated PDL cells. Notably, a CTSS upregulation by proinflammatory cytokines has also been found in smooth muscle cells, blood vessel cells, and those in the cervix [25].

Interestingly, our experiments revealed that the stimulatory effects of IL-1*β* and *F. nucleatum* were dependent on the MEK1/2 and JNK signaling pathways. The upregulated CTSS expression under inflammatory or microbial conditions was nearly abolished by preincubation of cells with specific inhibitors against MEK1/2 and JNK, indicating that the MAPK pathway plays an important part in mediating the effects of inflammatory mediators and bacteria on the CTSS expression.

In our *in vitro* experiments, only IL-1*β* was applied to mimic inflammatory conditions. However, periodontitis is a complex inflammatory disease, and a great number of inflammatory mediators contribute to tissue destruction and bone loss. Further studies should clarify whether other inflammatory mediators cause similar responses by periodontal cells. Furthermore, periodontitis represents a polymicrobial disease and is caused by a complex subgingival biofilm. In addition to *F. nucleatum*, other bacteria are involved in the initiation and progression of periodontitis [52]. Moreover, PDL cells were exposed to lysate from nonvital *F. nucleatum* in our experiments, suggesting that LPS might have been a main contributor to the observed effects. However, other virulence factors might have also participated in the stimulation of CTSS synthesis. Further investigations should therefore address the potential of other bacteria, vital and nonvital, for the induction of CTSS in periodontal cells.

To study the synthesis and regulation of CTSS in periodontal cells in a more complex environment, gingival biopsies from rats with ligature-induced periodontitis were analyzed. As recently shown by our group [39], the ligature-supported plaque accumulation caused significant periodontal inflammation and bone loss. By using this animal model, it was possible to study the CTSS expression in periodontal tissues under realistic conditions. But even more important, it was possible to follow the time course of the aforementioned CTSS expression in this complex environment. Our *in vivo* results showed significantly higher CTSS expression levels in gingival biopsies from sites of periodontal inflammation over 12 days, as compared to periodontally healthy sites. These results not only confirm but also expand our *in vitro* data, demonstrating that inflammatory and microbial insults upregulate CTSS in periodontal cells.

In order to extrapolate our in vivo data from rat gingival biopsies to humans, we also collected gingival biopsies from subjects with periodontal diseases and periodontally healthy individuals [37]. Like biopsies from the ligature-induced periodontitis, human gingival tissues from sites of periodontitis also exhibited increased CTSS expression levels. Moreover, as visualized by immunohistochemistry, enhanced CTSS protein staining was also detected in biopsies of periodontitis patients. CTSS staining was mainly found in gingival fibroblast and macrophages but not in epithelial cells. These findings confirmed our data from the *in vitro* experiments, which demonstrated that fibroblastic cells synthesize CTSS, and expanded these results by showing which other cells might contribute to CTSS expression in the periodontium.

In our *in vitro* experiments, PDL cells, which constitute a heterogeneous cell population, were used, because they are critical for both periodontal destruction and regeneration. The cells were phenotyped before our experiments, confirming their ability to differentiate into osteoblastic cells. Since no osteogenic medium was applied in our *in vitro* experiments, these cells attained a fibroblastic phenotype, which facilitated the comparisons with the gingival biopsies from human and rats.

Very little is known about the link between CTSS and periodontal diseases. Recently, it was suggested by Gonzalez et al. [53] who studied the gene expression in gingival biopsies from rhesus monkeys that CTSS might play a role in periodontitis, which supports strongly our findings at transcriptional and protein levels in human periodontal cells and tissues. Furthermore, CTSS was identified as a possible periodontitis-related target in a bioinformatics-based approach [30]. In this study, gene expression differences between periodontally healthy and diseased sites of periodontitis patients were analyzed. It was suggested that CTSS might be a hub protein which plays a central role in periodontal bone loss, thereby also supporting our *in vitro* and *in vivo* results.

Although we have shown that CTSS is produced by PDL cells and increased under inflammatory and infectious conditions, the functional role of this autophagy-associated molecule in periodontal cells and tissues has yet to be elucidated. Interestingly, it has been shown that CTSS can inhibit autophagy, which is also a process which can ensure cell survival [13, 21]. Therefore, regulation of CTSS might be a critical pathogenic mechanism in periodontitis, which should be investigated in further studies.

## 5. Conclusions

In conclusion, we provide substantial evidence that CTSS is expressed in periodontal cells and tissues under inflammatory and microbial conditions. CTSS may be an important player in the initiation and progression of periodontal diseases.

## Figures and Tables

**Table 1:** Regulation of autophagy-associated genes by IL-1*β* at 1 d.

Target gene	Control (normalized expression)	IL-1*β* (normalized expression)	Regulation of expression (fold of control)
CTSS	0.02147	0.04437	2.06698
DRAM1	0.74956	1.13656	1.51630
BCL2	0.00884	0.00563	−1.56975
ATG3	0.52789	0.33625	−1.56992
FAS	0.22418	0.13826	−1.62145
TMEM74	0.01275	0.00719	−1.77425
DAPK1	0.01713	0.00908	−1.88681
ESR1	0.01500	0.00755	−1.98755
CXCR4	0.00373	0.00168	−2.22252
IRGM	0.01824	0.00719	−2.53548

**Table 2:** Regulation of autophagy-associated genes by *F. nucleatum* at 1 d.

Target gene	Control (normalized expression)	*F. nucleatum* (normalized expression)	Regulation of expression (fold of control)
CTSS	0.02147	0.09855	4.59035
PIK3CG	0.00001	0.00004	4.03971
DRAM1	0.74956	1.92932	2.57393
IGF1	0.01431	0.03249	2.27069
NFKB1	0.40746	0.85589	2.10054
BID	0.14601	0.30644	2.09878
BAK1	0.01303	0.02353	1.80636
TNFSF10	0.00721	0.01299	1.80182
INS	0.00960	0.01558	1.62314
TMEM74	0.01275	0.01934	1.51678

## References

[1] Thornton-Evans G., Eke P., Wei L. (2013). Periodontitis among adults aged ≥30 years - United States, 2009-2010. *MMWR Supplements*.

[2] Marchesan J. T., Scanlon C. S., Soehren S., Matsuo M., Kapila Y. L. (2011). Implications of cultured periodontal ligament cells for the clinical and experimental setting: a review. *Archives of Oral Biology*.

[3] Konermann A., Stabenow D., Knolle P. A., Held S. A. E., Deschner J., Jäger A. (2012). Regulatory role of periodontal ligament fibroblasts for innate immune cell function and differentiation. *Innate Immunity*.

[4] Pihlstrom B. L., Michalowicz B. S., Johnson N. W. (2005). Periodontal diseases. *The Lancet*.

[5] Sbordone L., Bortolaia C. (2003). Oral microbial biofilms and plaque-related diseases: microbial communities and their role in the shift from oral health to disease. *Clinical Oral Investigations*.

[6] Tatakis D. N., Kumar P. S. (2005). Etiology and pathogenesis of periodontal diseases. *Dental Clinics of North America*.

[7] Yucel-Lindberg T., Båge T. (2013). Inflammatory mediators in the pathogenesis of periodontitis. *Expert Reviews in Molecular Medicine*.

[8] Chávarry N. G., Vettore M. V., Sansone C., Sheiham A. (2009). The relationship between diabetes mellitus and destructive periodontal disease: a meta-analysis. *Oral Health & Preventive Dentistry*.

[9] Chistiakov D. A., Orekhov A. N., Bobryshev Y. V. (2016). Links between atherosclerotic and periodontal disease. *Experimental and Molecular Pathology*.

[10] Kroemer G., Mariño G., Levine B. (2010). Autophagy and the integrated stress response. *Molecular Cell*.

[11] King J. S., Veltman D. M., Insall R. H. (2011). The induction of autophagy by mechanical stress. *Autophagy*.

[12] Ma Y., Galluzzi L., Zitvogel L., Kroemer G. (2013). Autophagy and cellular immune responses. *Immunity*.

[13] Mariño G., Niso-Santano M., Baehrecke E. H., Kroemer G. (2014). Self-consumption: the interplay of autophagy and apoptosis. *Nature Reviews Molecular Cell Biology*.

[14] Glick D., Barth S., Macleod K. F. (2010). Autophagy: cellular and molecular mechanisms. *The Journal of Pathology*.

[15] Lapaquette P., Guzzo J., Bretillon L., Bringer M. A. (2015). Cellular and molecular connections between autophagy and inflammation. *Mediators of Inflammation*.

[16] Quan W., Lim Y. M., Lee M. S. (2012). Role of autophagy in diabetes and endoplasmic reticulum stress of pancreatic β-cells. *Experimental & Molecular Medicine*.

[17] Bullon P., Cordero M. D., Quiles J. L. (2012). Autophagy in periodontitis patients and gingival fibroblasts: unraveling the link between chronic diseases and inflammation. *BMC Medicine*.

[18] Huang W. P., Klionsky D. J. (2002). Autophagy in yeast: a review of the molecular machinery. *Cell Structure and Function*.

[19] Wesselborg S., Stork B. (2015). Autophagy signal transduction by ATG proteins: from hierarchies to networks. *Cellular and Molecular Life Sciences*.

[20] Huang C. C., Lee C. C., Lin H. H., Chen M. C., Lin C. C., Chang J. Y. (2015). Autophagy-regulated ROS from xanthine oxidase acts as an early effector for triggering late mitochondria-dependent apoptosis in cathepsin S-targeted tumor cells. *PLoS One*.

[21] Zhang L., Wang H., Xu J., Zhu J., Ding K. (2014). Inhibition of cathepsin S induces autophagy and apoptosis in human glioblastoma cell lines through ROS-mediated PI3K/AKT/mTOR/p70S6K and JNK signaling pathways. *Toxicology Letters*.

[22] Shi G. P., Webb A. C., Foster K. E. (1994). Human cathepsin S: chromosomal localization, gene structure, and tissue distribution. *Journal of Biological Chemistry*.

[23] Petanceska S., Canoll P., Devi L. A. (1996). Expression of rat cathepsin S in phagocytic cells. *Journal of Biological Chemistry*.

[24] Zavanik-Bergant V., Sekirnik A., Golouh R., Turk V., Kos J. (2001). Immunochemical localisation of cathepsin S, cathepsin L and MHC class II-associated p41 isoform of invariant chain in human lymph node tissue. *Biological Chemistry*.

[25] Dickinson D. P. (2002). Cysteine peptidases of mammals: their biological roles and potential effects in the oral cavity and other tissues in health and disease. *Critical Reviews in Oral Biology & Medicine*.

[26] Nakanishi H. (2003). Microglial functions and proteases. *Molecular Neurobiology*.

[27] Andrault P. M., Samsonov S. A., Weber G. (2015). Antimicrobial peptide LL-37 is both a substrate of cathepsins S and K and a selective inhibitor of cathepsin L. *Biochemistry*.

[28] Pietschmann P., Foger-Samwald U., Sipos W., Rauner M. (2013). The role of cathepsins in osteoimmunology. *Critical Reviews in Eukaryotic Gene Expression*.

[29] Garg G., Pradeep A. R., Thorat M. K. (2009). Effect of nonsurgical periodontal therapy on crevicular fluid levels of cathepsin K in periodontitis. *Archives of Oral Biology*.

[30] Song L., Yao J., He Z., Xu B. (2015). Genes related to inflammation and bone loss process in periodontitis suggested by bioinformatics methods. *BMC Oral Health*.

[31] Mariotti A., Cochran D. L. (1990). Characterization of fibroblasts derived from human periodontal ligament and gingiva. *Journal of Periodontology*.

[32] Basdra E. K., Komposch G. (1997). Osteoblast-like properties of human periodontal ligament cells: an *in vitro* analysis. *European Journal of Orthodontics*.

[33] Nokhbehsaim M., Winter J., Rath B., Jäger A., Jepsen S., Deschner J. (2011). Effects of enamel matrix derivative on periodontal wound healing in an inflammatory environment in vitro. *Journal of Clinical Periodontology*.

[34] Nokhbehsaim M., Eick S., Nogueira A. V. B. (2013). Stimulation of MMP-1 and CCL2 by NAMPT in PDL cells. *Mediators of Inflammation*.

[35] Nokhbehsaim M., Keser S., Nogueira A. V. B. (2014). Beneficial effects of adiponectin on periodontal ligament cells under normal and regenerative conditions. *Journal of Diabetes Research*.

[36] Nokhbehsaim M., Keser S., Nogueira A. V. (2014). Leptin effects on the regenerative capacity of human periodontal cells. *International Journal of Endocrinology*.

[37] Damanaki A., Nokhbehsaim M., Eick S. (2014). Regulation of NAMPT in human gingival fibroblasts and biopsies. *Mediators of Inflammation*.

[38] Nogueira A. V. B., Nokhbehsaim M., Eick S. (2014). Regulation of visfatin by microbial and biomechanical signals in PDL cells. *Clinical Oral Investigations*.

[39] Nogueira A. V. B., de Molon R. S., Nokhbehsaim M., Deschner J., Cirelli J. A. (2017). Contribution of biomechanical forces to inflammation-induced bone resorption. *Journal of Clinical Periodontology*.

[40] Hsing L. C., Rudensky A. Y. (2005). The lysosomal cysteine proteases in MHC class II antigen presentation. *Immunological Reviews*.

[41] Hou W. S., Li W., Keyszer G. (2002). Comparison of cathepsins K and S expression within the rheumatoid and osteoarthritic synovium. *Arthritis & Rheumatology*.

[42] Rauner M., Föger-Samwald U., Kurz M. F. (2014). Cathepsin S controls adipocytic and osteoblastic differentiation, bone turnover, and bone microarchitecture. *Bone*.

[43] Hsieh M. J., Lin C. W., Chen M. K. (2017). Inhibition of cathepsin S confers sensitivity to methyl protodioscin in oral cancer cells via activation of p38 MAPK/JNK signaling pathways. *Scientific Reports*.

[44] Arnlöv J. (2012). Cathepsin S as a biomarker: where are we now and what are the future challenges?. *Biomarkers in Medicine*.

[45] Jobs E., Risérus U., Ingelsson E. (2013). Serum cathepsin S is associated with decreased insulin sensitivity and the development of type 2 diabetes in a community-based cohort of elderly men. *Diabetes Care*.

[46] Fuggle N. R., Smith T. O., Kaul A., Sofat N. (2016). Hand to mouth: a systematic review and meta-analysis of the association between rheumatoid arthritis and periodontitis. *Frontiers in Immunology*.

[47] Papageorgiou S. N., Reichert C., Jäger A., Deschner J. (2015). Effect of overweight/obesity on response to periodontal treatment: systematic review and a meta-analysis. *Journal of Clinical Periodontology*.

[48] Signat B., Roques C., Poulet P., Duffaut D. (2011). *Fusobacterium nucleatum* in periodontal health and disease. *Current Issues in Molecular Biology*.

[49] He J., Huang W., Pan Z. (2011). Quantitative analysis of microbiota in saliva, supragingival, and subgingival plaque of Chinese adults with chronic periodontitis. *Clinical Oral Investigations*.

[50] Sina C., Lipinski S., Gavrilova O. (2012). Extracellular cathepsin K exerts antimicrobial activity and is protective against chronic intestinal inflammation in mice. *Gut*.

[51] Müller S., Faulhaber A., Sieber C. (2014). The endolysosomal cysteine cathepsins L and K are involved in macrophage-mediated clearance of Staphylococcus aureus and the concomitant cytokine induction. *The FASEB Journal*.

[52] Hajishengallis G. (2015). Periodontitis: from microbial immune subversion to systemic inflammation. *Nature Reviews Immunology*.

[53] Gonzalez O. A., Novak M. J., Kirakodu S. (2014). Comparative analysis of gingival tissue antigen presentation pathways in ageing and periodontitis. *Journal of Clinical Periodontology*.

